# Serological and molecular detection of bovine leukemia virus in cattle in Iraq

**DOI:** 10.1038/emi.2016.60

**Published:** 2016-06-08

**Authors:** Yahia Ismail Khudhair, Saleem Amin Hasso, Nahi Y Yaseen, Ahmed Majeed Al-Shammari

**Affiliations:** 1 Department of Veterinary Medicine, College of Veterinary Medicine, Al-University of Qadisiyah, Al Diwaniyah 58002, Iraq; 2 Department of Veterinary Medicine, College of Veterinary Medicine, University of Baghdad, Baghdad 10001, Iraq; 3 Experimental Therapy Department, Iraqi Center for Cancer and Medical Genetic Research, Mustansiriyah University, Baghdad 10001, Iraq

**Keywords:** enzootic bovine leucosis, Iraq, persistent lymphocytosis

## Abstract

Bovine leukemia virus (BLV) is highly endemic in many countries, including Iraq, and it impacts the beef and dairy industries. The current study sought to determine the percentage of BLV infection and persistent lymphocytosis (PL) in cattle in central Iraq. Hematological, serological, and molecular observations in cross breeds and local breeds of Iraqi cattle naturally infected with BLV were conducted in the peripheral blood mononuclear cells of 400 cattle (340 cross breed and 60 local breed) using enzyme-linked immunosorbent assay and polymerase chain reaction (PCR). On the basis of the absolute number of lymphocytes, five of the 31 positive PCR cases had PL. Among these leukemic cattle, one case exhibited overt neutrophilia. Serum samples were used to detect BLV antibodies, which were observed in 28 (7%) samples. PCR detected BLV provirus in 31 samples (7.75%). All 28 of the seropositive samples and the 3 seronegative samples were positive using PCR. Associations were observed between bovine leukosis and cattle breed, age and sex. Age-specific analysis showed that the BLV percentage increased with age in both breeds. Female cattle (29 animals; 7.34%) exhibited significantly higher infectivity than male cattle (two animals; 4.34%). In conclusion, comprehensive screening for all affected animals is needed in Iraq; programs that segregate cattle can be an effective and important method to control and/or eliminate the BLV.

## Introduction

Enzootic bovine leukosis (EBL) is the most important type of bovine lymphotropic retrovirus infection, and this disease is caused by bovine leukemia virus (BLV).^1, 2^ According to the International Committee for Taxonomy of Viruses classification scheme, BLV is a deltaretrovirus genus of the retroviridae family.^3^ BLV has many structural and functional characteristics common with human T-lymphotropic viruses.^4^ BLV causes chronic infection in cattle and develops into three possible pathological forms. Most BLV-infected animals appear asymptomatic (clinically healthy).^5^ Approximately one-third of infected animals develop persistent lymphocytosis (PL) due to polyclonal proliferation of B lymphocytes, and 0.1%–10% develop lymphoid tumors, primarily B-cell lymphosarcomas.^6, 7^

BLV affects the health of infected animals and impacts the beef and dairy industries.^8^ BLV infects approximately one-third of the adult dairy cattle in the United States, and is a major cause of the loss of export markets for breeding cattle.^9^ Direct economic losses are incurred because of death, decreases in milk productivity, fertility and life span, and condemnation at slaughter.^8, 10^

BLV is widespread in populations of cattle worldwide, and infection remains endemic in many countries.^11^ BLV has high prevalence in South America, some Asiatic and Middle Eastern countries, and Eastern Europe.^12, 13, 14, 15^

The most important source of BLV transmission from infected cattle is via blood lymphocytes and other tissue products.^16^ Close contact transmission, hematophagous flies and iatrogenic transfer through the use of contaminated veterinary instruments are all well-documented sources of BLV transmission between infected and non-infected cattle.^17, 18, 19^ Although in utero transmission of BLV from a cow to its fetus can occur, it is relatively rare.^20^ Colostrum, milk-borne and artificial insemination BLV transmission have also been reported.^20, 21, 22^

Agar gel immunodiffusion is the prescribed diagnostic test for international trade and the most common method used to detect BLV-specific antibodies. Several enzyme-linked immunosorbent assays (ELISA) are also available to diagnose BLV.^23, 24^ Agar gel immunodiffusion is as sensitive as the routinely used indirect ELISA test; it is highly specific, reliable and easy to perform.^25^ PCR has also been described as a diagnostic test.^26^ PCR is a useful method to detect recently infected animals before seroconversion. It can also be used to confirm neonatal BLV infection because serological tests cannot differentiate between antibodies produced *de novo* in response to infection and those that are transferred passively in colostrum.^27, 28^

The prevalence of BLV worldwide varies widely between countries; prevalence has been found to be as low as 5% in Cambodia and Taiwan^19^ and 17% in Turkey^29^ or as high as 83.9% in the US and 25.7% in Canada.^30, 31^ Although BLV impacts the Iraqi economy because it is neglected in Iraq, this study sought to identify a comprehensive molecular and seroepidemiological screening for infected animals to establish a provision for disease control and eradication.

## Materials and methods

### Ethics statement

The Baghdad University College of Veterinary Medicine Review Board and Institutional Review Board of the Iraqi Center for Cancer and Medical Genetic research approved this study. Consent was obtained from the farm owners before animal sampling.

### Animals

The animals examined were dairy cattle raised on private dairy farms located in two governorates, Al Qadisiyah and Al Mouthanna, as well as animals of one station dairy. A total of 400 cross-breed cattle (Friesian with native cattle) and local-breed cattle (native cattle) were investigated. The samples were divided into 227, 78 and 95 cattle from the Al Qadisiyah, Al Mouthanna and station dairy herds, respectively. The cattle were older than six months and were selected based on clinical signs indicating that they may had BLV. The cattle were divided into two age groups: ⩾two years old and <two years old. The study began in March 2014 and was completed in December 2015.

### Sample collection

Registered veterinarians obtained peripheral blood aseptically from the jugular vein with and without anticoagulant using a vacutainer system in two sterile vacuum tubes. The samples were then refrigerated and stored until they arrived at the laboratory.

### Hematological examination

Fresh peripheral blood samples were used for total leukocyte and differential leukocyte counts, and obtained by standard veterinary procedures.^32^ The leukocyte count was performed manually using a hemocytometer counting chamber to determine the number of leukocytes per 1 μL of blood. The differential leukocyte count was performed using a thin blood smear stained with a Diff-Quik stain kit (Syrbio, Switzerland), and a count of 200 atypical leukocytes was considered as being a positive case.

### Serum preparation and serological test

The blood samples were centrifuged for 10 min at 2000 rpm, and then, serum was collected in 1.5 mL Eppendorf tubes and stored at −80 °C until further analysis. All 400 serum samples were tested for BLV using a Svanovir BLV-gp51-Ab ELISA test kit (Svanova Biotech AB, Uppsala, Sweden). The procedures were performed according to the manufacturer's instructions.

### Polymerase chain reaction (PCR)

Genomic DNA was extracted from peripheral blood mononuclear cells (PBMCs) after isolation from whole blood using the Histopaque 1077 density gradient technique (Sigma-Aldrich, Steinheim, Germany). DNA extraction was performed using a Magnesia Genomic DNA Whole Blood Kit and Magnesia Automated DNA Extraction machine (Anatolia Geneworks, İstanbul, Turkey). All DNA extraction steps were performed according to the manufacturer's instructions. The extracted DNA samples were quantified and stored at −86 °C until used.

The extracted DNA samples were used as a template to detect BLV proviral DNA by single PCR using two sets of primers, a pol1 primer set and an env1 primer set. In the pol1 primer, the forward primer was 5′-CGG GAT TGA TCA CCC CGG AA-3 (546–565), and the reverse primer was 5′-GGA CTC CGT CGG GAA GGT T-3 (1033–1052). These were based on conserved regions of the 3′ end of the *pol* gene using an online program (OligoAnalyzer 3.1, Integrated DNA Technologies, Inc., Coralville, IA, USA) to amplify a 507-bp fragment.

The reaction final volume was 25 μL, which consisted of 12.5 μL KAPA2G of Robust HotStart ReadyMix 1 × (Kapa Biosystems, Cape Town, South Africa) containing 0.2 mM/L of each dNTP, 3 mM/L of MgCl_2_, 1 unit of Robust HotStart DNA polymerase, 1.5 μL (0.6 μM) of each primer, 4 μL of the extracted DNA samples and 5.5 μL of PCR-grade water. Using a SureCycler 8800 Thermo Cycler (Agilent technologies, Santa Clara, CA, USA), the theromocycling conditions were as follows: initial denaturation at 95 °C for 5 min, followed by 35 cycles of denaturation at 95 °C for 20 s, annealing at 61 °C for 20 s and extension at 69 °C for 1 min, with a final extension at 69 °C for 3 min. The second primer set for the *env1* gene was based on previous publications.^33, 34^ In the *env1* set, the forward primer was 5-CCC ACA AGG GCG GCG CCG GTT T-3 (5099–5120), and the reverse primer 5-GCG AGG CCG CGT CCA GAG CTG G-3 (5521–5542). The PCR mixture (25 μL) consisted of 12.5 μL of 1 μ polymerase buffer, 2 mM MgCl_2_, 0.2 of each mM dNTP, 1 μL of 0.5 mM of each primer, 4 μL of 0.5 mg of DNA and 7.5 μL of PCR-grade water. Amplification conditions started with initial denaturation at 94 °C for 3 min, followed by 35 cycles of denaturation at 94 °C for 1 min, primer annealing at 65 °C for 20 s and elongation at 72 °C for 30 s. The final elongation of amplification was 5 min.

The final amplified products were detected by electrophoresis through a 1% agarose gel containing ethidium bromide in Tris/Borate/EDTA buffer (90 mM Tris-borate and 2 mM EDTA). DNA was visualized using VISION Gel Documentation (Scie-Plas, Cambridge, UK).

## Results

### Clinical findings

Most BLV-infected cattle were clinically asymptomatic during examination and exhibited only nonspecific findings, such as emaciation, rough hair coat and pale mucus membrane.

### Hematological findings

According to cattle age, an absolute lymphocyte count of more than 8000 lymphocytes/μL was diagnosed as leukemic leukosis (persistent lymphocytosis: PL). Of the 31 BLV-positive cattle, the total leukocyte counts of five animals (29.0%) were 16 950, 10 900, 15 000, 29 250 and 15 950 lymphocytes/μL. The absolute lymphocytes count for these samples were 8390, 8271, 9636, 21 090 and 10 148 lymphocytes/μL, respectively; thus, PL was diagnosed. Based on the European hematological diagnostic guidelines (Table 1) one sample with a serological reaction had neutrophilia with increased band cells, whereas the remaining samples (22 of 31; 70.9%) were within normal limits. A small number of atypical lymphocytes were observed in the some BLV-positive cattle. No hematologic evidence of bovine leukosis was observed in the BLV-negative cattle, but cattle samples with leukocytosis with lymphocytosis or neutrophilia and whose serological and molecular results were negative were excluded. Leukosis in these animals might have been due to other diseases, such as blood parasites (theileriosis and anaplasmosis), which had appeared in the blood smears of some of these cattle.

Serum antibodies against BLV were detected in 28 (7%) of the 400 samples. The seroprevalence rates of BLV in the three location areas were 8.8% for the Al Qadisiyah herd, 2.6% for the Al Mouthanna herd and 6.3% for the station dairy herd (Table 2). These sample locations were significantly different (*P*=0.01) (χ^2^ test).

The serum samples exhibited varying degrees of reactivity in the ELISA; of the 28 seropositive samples, seven exhibited a strong positive reaction, and the remaining 21 samples reacted weakly.

### Polymerase chain reaction (PCR)

**Figure 1:**
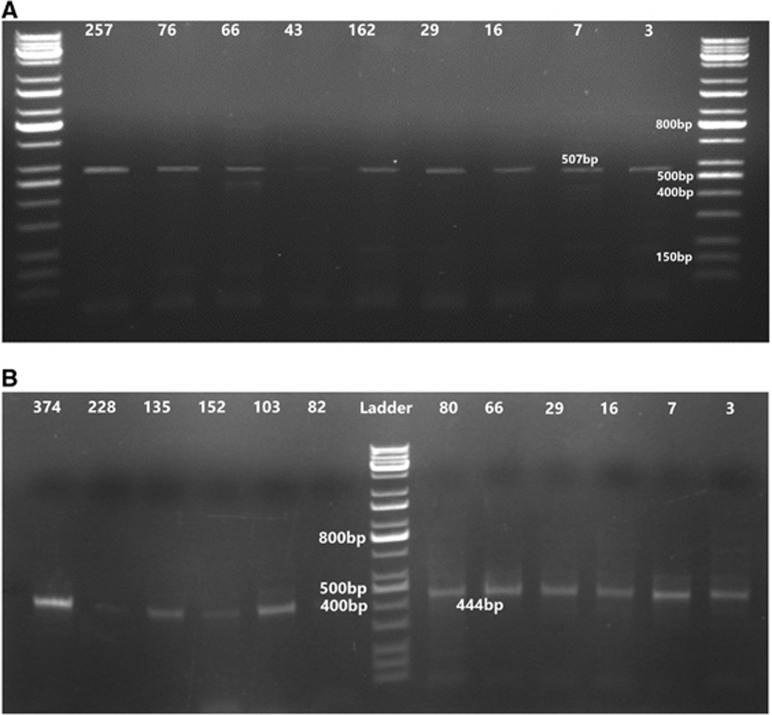
Ethidium bromide-stained agarose gel electrophoresis of the PCR product of BLV. (**A**) Loading 15 μL of the amplification resulted in the predicted 507-bp amplification product of the *pol* gene of the BLV provirus through 1% agarose stained with ethidium bromide. (**B**) PCR product of BLV (444-bp fragment of the *env* gene) in positive PBMCs isolated from the blood samples. Bovine leukemia virus, BLV; peripheral blood mononuclear cells, PBMCs; polymerase chain reaction, PCR.

Using two sets of specific BLV primers, the PCR results confirmed the BLV serological results. Of the 400 examined DNA samples, 31 (7.75%) were as positive, and all seropositive samples that underwent PCR analysis tested positive for both primer sets. In addition, the three (0.8%) samples seronegative with ELISA were positive using PCR (Table 3). The PCR results confirmed the presence of 444-bp and 507-bp fragments of the *pol1* and *env1* genes, which were amplified to the standard ladder bands (Figure 1).

The epidemiological results are summarized in Table 4. The highest percentage (8.3%) of infection was observed among the local breed of cattle (five animals); in the cross-breed cattle, the percentage of infection (7.6%) was slightly lower (26 animals) (*P*⩽0.05). Among the two age groups, only two of the 70 cattle in the <two years old group were positive, whereas 29 of the 330 cattle (8.8%) in the ⩾two years-old-age group were positive; this difference was significant (*P*⩾0.05). The females exhibited a higher percentage of infection (7.34% 29 animals) compared with males (4.34%, two animals).

## Discussion

This study is the first in the middle Euphrates region in Iraq to confirm the presence of BLV in all cattle breeds and to describe its epidemiology. The percentage of BLV infection was 7.75% in the Iraqi cattle examined here, as measured by PCR. In neighboring countries, the percentage of BLV infection is 17% in Iran,^35^ 11% in Turkey^36^ and Jordon.^37^ The present study demonstrated that the percentage of BLV infection in Iraqi cattle is lower than other countries, such as Korea (35%),^38^ Tanzania (36%),^39^ China (21.24%)^40^ and Thailand (32.5%),^41^ whereas the percentage in Iraq was higher than the 3.67% reported in some parts of Turkey.^42^ Differences in the percentage of BLV infection are likely to occur between countries and locations within the same country.

A previous serological study in Baghdad, Iraq, reported BLV infection frequency very similar to that reported here (7%).^43^ The prevalence of infection observed here does not necessarily represent the actual prevalence of BLV infection in the studied areas because most samples were not randomly collected; instead, they were selected based on clinical signs that might indicate that the animal most likely infected with BLV.

The low occurrence of infection among Iraqi cattle might be related to certain conditions and management practices in the dairies investigated here because herd size has an important role in BLV prevalence.^44^ High prevalence of BLV in cattle is mostly related to high-density cattle populations and poor sanitation conditions; close physical contact and contaminated biological materials appear to be required for BLV transmission.^45^

Recent studies have shown that BLV sequences, which can be classified into seven distinct genotypes, are circulating in cattle from the US and South America.^46^ Weak reactions using ELISA indicated a high percentage (67.8%) of seropositive samples, and most weak reactivity is likely due to the variable specificity of the serum antibodies or the presence of preexisting low levels of antibodies due to a latent natural infection in these animals.^47^ The low reactivity could also be because the serum antibodies possess low-affinity constants, which can lead to dissociation of the antigen–antibody complex during the multiple washing steps of the assay and consequently result in a weak positive reactivity in some cases. In addition, the probability of different antigenic strains of BLV with relative immunogenicity is characteristic of weak reaction using serological tests.^48^

Serological tests have been used more extensively to identify BLV-infected cattle worldwide due to their rapidity, cost-effectiveness and easy interpretation.^46^ BLV status conversion is detected via PCR assay more rapidly than via ELISA in recent infections, before the development of antibodies, in doubtful reactions, and weak positive reactions in ELISA.^26^ Thus, PCR amplification was used to examine seropositive cattle and seronegative cattle for the presence of BLV provirus in PBMCs.

The provirus integration of BLV has been investigated in PBMCs isolated from cattle blood.^49^ In the present study, PCR use in the preliminary field screen further demonstrated the advantages of PCR as a BLV detection technique. PCR was performed based on primer sites within conserved regions of the *pol* and *env* genes that flank a region of variability. We hypothesized that by basing the primer design on the conserved regions, the assay would be able to detect a variety of serologically different BLV strains.^50^ BLV infection in cattle results in a strong permanent antibody response to the BLV antigens weeks after infection, and some infected cattle may carry the provirus and not have detectable antibody titers.^32^ All serologically positive samples and the three negative samples were positive using PCR; thus, our results are in close agreement to those reported in Brandon *et al.*,^51^ which noted that the sensitivity of PCR permitted the detection of bovine leukemia provirus in 6.8% of serologically negative BLV-exposed cattle.

Leukocyte counts in some of the BLV-infected cows were significantly higher due to significantly higher lymphocyte counts, which has also been described in a study that found 8800 (56%) to 9595 (67.5%) of the BLV-infected cows had PL.^52^

Weight loss, poor hair coat and weakness are the clinical signs most commonly associated with BLV infection in cattle. In the present study, these abnormal clinical signs were recorded during the animal examination and sample collection, and our findings were similar to observations by others.^52^

The present study also demonstrated an association between age and BLV infection. Infection most commonly occurred in animals >two years old, and the incidence of infection increased with age based on virus detection in colostrum and milk, suggesting a role of both colostrum and milk in disease transmission.^52, 53^ The higher prevalence of BLV in older animals could be caused by several different factors. When animals are housed in the same free-stall barns, close contact among animals could increase transmission. As a cow ages, the likelihood of sufficient contact with an infected animal and transmission of the infection from infected herd mates increases. In addition, older animals are more susceptible to infections.^4^ These results were similar to those of other studies.^35, 51^ The present study provides evidence that BLV infection in Iraqi cattle is endemic. More attention to this disease is required to establish effective prevention and control measures. A comprehensive screening of all animals should be conducted on all Iraq cattle farms. The programs to segregate infected animals and eliminate transmission can be effective and particularly important for controlling the spread of BLV because no effective vaccines are available.^54, 55^

## Figures and Tables

**Table 1:** Summary of the BLV-positive samples with the three methods

**Cattle sample**	**Sex**	**Age (years)**	**WBC (μL)**	**Lymphocyte (μL)**	**Hematology**	**ELISA**	**PCR**
3	F	6	16950	8390	PL	+	+
7	F	7	11650	6638	AL	+	+
16	F	9	10900	8271	PL	+	+
29	F	8	11900	6188	AL	+	+
43	F	10	8500	6946	AL	+	+
66	F	6	22000	7946	AL	−	+
76	F	7	11750	4582	AL	+	+
80	F	8	19550	5980	AL	+	+
82	F	6	12850	5810	AL	+	+
103	F	8	7600	3990	AL	+	+
135	M	2.5	22850	10800	AL	+	+
139	F	7	10250	7412	AL	+	+
152	F	7	7500	4010	AL	+	+
158	F	12	15750	6636	AL	−	+
162	F	8	15000	9675	PL	+	+
166	F	12	4250	2016	AL	+	+
170	F	1.5	8200	3840	AL	+	+
181	F	8	5950	3098	AL	+	+
194	F	7	9850	4688	AL	+	+
200	F	10	5426	1899	AL	+	+
228	F	7	61750	5372/ (41002 neutrophilia)	Neutrophilia	+	+
253	F	2	8200	5125	AL	−	+
257	F	5	29250	21090	PL	+	+
279	F	4	24700	7113	AL	+	+
297	F	5	12950	6410	AL	+	+
299	F	4	16400	4740	AL	+	+
303	M	4	13150	6910	AL	+	+
331	F	16	3800	844	AL	+	+
340	F	7	6800	4420	AL	+	+
364	F	10	11600	5498	AL	+	+
374	F	8	15950	10148	PL	+	+

Abbreviations: persistent lymphocytosis, PL; aleukemic leukemia, AL.

**Table 2:** Distribution of seropositive cattle in the sampled study areas

**City**	**Infected**	**Non-infected**	**Total**
Al Qadisiyah	20 (8.8%)	205	225
Al Mouthanna	2 (2.6%)	76	78
Station herd	6 (6.3%)	91	97
Total	28 (7%)	372	400

**Table 3:** BLV infectivity percentages by ELISA and PCR examination of 400 cattle samples

**Test**	**PCR+**	**PCR−**	**Total**
ELISA+	28 (7%)	0	28 (7%)
ELISA−	3 (0.75%)	369	372
Total	31(7.75%)	369	400

**Table 4:** Identification of BLV infection according to breed, age and sex

	**Cattle breed**			**Age**			**Sex**		
	**Cross breed**	**Local breed**	**Total**	**⩾2 year**	**<2 year**	**Total**	**Male**	**Female**	**Total**
Infected	26 (7.6%)	5 (8.3%)	31 (7.75%)	2 (2.85%)	29 (8.8%)	31 (7.75%)	2 (4.34%)	29 (7.34%)	31 (7.75%)
Uninfected	314	55	369	68	301	869	44	325	369
Total	340	60	400	70	330	400	46	354	400

## References

[bib1] Kettmann R, Burny A, Callebaut I et al. Bovine leukemia virus. In: Levy JA (ed). The Retroviridae. New York: Plenum Press; 1994, pp 39–81.

[bib2] Rovnak J, Casey JW, Boyd AL et al. Isolation of bovine leukemia virus infected endothelial cells from cattle with persistent lymphocytosis. Lab Invest 1991; 65: 192–202. 1652665

[bib3] King AM, Adams MJ, Carstens EB et al. Virus Taxonomy. Ninth Report of the International Committee on Taxonomy of Viruses. London: Elsevier. 2012, pp 486–487.

[bib4] Burny A, Bruck C, Cleuter Y et al. Bovine leukemia virus, a distinguished member of the human T-lymphotropic virus family. Princess Takamatsu Symp 1984; 15: 219–227. 6100641

[bib5] Trono KG, Perez-Filgueira DM, Duffy S et al. Seroprevalence of bovine leukemia virus in dairy cattle in Argentina: comparison of sensitivity and specificity of different detection methods. Vet Microbiol 2001; 83: 235–248. 11574172 10.1016/s0378-1135(01)00420-5

[bib6] Schwartz I, Levy D. Pathobiology of bovine leukemia virus. Vet Res 1994; 6: 521–536. 7889034

[bib7] Pyeon D, O'Reilly KL, Splitter GA. Increased interleukin-10 mRNA expression in tumor-bearing or persistently lymphocytotic animals infected with bovine leukemia virus. J Virol 1996; 70: 5706–5710. 8764093 10.1128/jvi.70.8.5706-5710.1996 PMC190539

[bib8] Ott SL, Johnson R, Wells SJ. Association between bovine-leukosis virus seroprevalence and herd-level productivity on US dairy farms. Prev Vet Med 2003; 61: 249–262. 14623410 10.1016/j.prevetmed.2003.08.003

[bib9] Erskine RJ, Bartlett PC, Byrem TM et al. Association between bovine leukemia virus, production, and population age in Michigan dairy herds. J Dairy Sci 2012; 95: 727–734. 22281337 10.3168/jds.2011-4760

[bib10] Kale M, Bulut O, Yapk O et al. Effects of subclinical bovine leukemia virus infection on some production parameters in a dairy farm in southern Turkey. J S Afr Vet Assoc 2007; 78: 130–132. 18237034 10.4102/jsava.v78i3.303

[bib11] Murtaugh MP, Lin GF, Haggard DL et al. Detection of bovine leukemia virus in cattle by the polymerase chain reaction. J Virol Methods 1991; 33: 73–85. 1658030 10.1016/0166-0934(91)90009-o

[bib12] Samara SI, Lima EG, Nascimento AA. Monitorinh of enzootic bovine leukosis in dairy cattle from the Pitangueiras region in Sao Paulo, Brazil. Braz J Vet Res Animal Sci 1997; 34: 349–351.

[bib13] Polat M, Ohno A, Takeshima SN et al. Detection and molecular characterization of bovine leukemia virus in Philippine cattle. Arch Virol 2015; 160: 285–296. 25399399 10.1007/s00705-014-2280-3

[bib14] Mohammadi V, Atyabi N, Nikbakht BG et al. Seroprevalence of bovine leukemia virus in some dairy farms in Iran. Global Veterinaria 2011; 7: 305–309.

[bib15] Rola-Luszczak M, Pluta A, Olech M et al. The molecular characterization of bovine leukaemia virus isolates from eastern Europe and Siberia and its impact on phylogeny. PLoS ONE 2013; 8: e58705. 23527009 10.1371/journal.pone.0058705 PMC3602460

[bib16] Mekata H, Sekiguchi S, Konnai S et al. Horizontal transmission and phylogenetic analysis of bovine leukemia virus in two districts of Miyazaki, Japan. J Vet Med Sci 2015; 77: 1115–1120. 25892699 10.1292/jvms.14-0624 PMC4591153

[bib17] Bech-Nielsen S, Piper CE, Ferrer JF. Natural mode of transmission of the bovine leukemia virus: role of bloodsucking insects. Am J Vet Res 1978; 39: 1089–1092. 209707

[bib18] Hopkins SG, Evermann JF, DiGiacomo RF et al. Experimental transmission of bovine leukosis virus by simulated rectal palpation. Vet Rec 1988; 122: 389–391. 2839925 10.1136/vr.122.16.389

[bib19] Meas S, Ohashi K, Tum S et al. Seroprevalence of bovine immunodeficiency virus and bovine leukemia virus in draught animals in Cambodia. J Vet Med Sci 2000; 62: 779–781. 10945301 10.1292/jvms.62.779

[bib20] Maaten J, Van der J, Miller M et al. In utero transmission of bovine leukemia virus. Am J Vet Res 1981; 42: 1052–1064. 6269468

[bib21] Buehring GC, Kramme PM, Schultz RD. Evidence for bovine leukemia virus in mammary epithelial cells of infected cows. Lab Invest 1994; 71: 359–365. 7933986

[bib22] Ferrer JF, Kenyon SJ, Gupta P. Milk of dairy cows frequently contains a leukemogenic virus. Science 1981; 213: 1014–1016. 6267692 10.1126/science.6267692

[bib23] Gonzalez ET, Junzo N, Alejandro RV et al. A rapid and sensitive diagnosis of bovine leukaemia virus infection using the nested shuttle polymerase chain reaction 1. Pesq Vet Bras 1999; 19: 63–1967.

[bib24] Simard C, Richardson S, Dixon P et al. Enzyme-linked immunosorbent assay for the diagnosis of bovine leukosis: comparison with the agar gel immunodiffusion test approved by the Canadian Food Inspection Agency. Can J Vet Res 2000; 64: 101–106. 10805248 PMC1189592

[bib25] Gonzlez ET, Bonzo EB, Echeverrla MG et al. Enzootic bovine leukosis: development of an indirect enzyme linked immunosorbent assay (I-ELISA) in serological studies. Rev Microbiol 1999; 30: 37–4.

[bib26] Evermann JF, Jackson MK. Laboratory diagnostic tests for retroviral infections in dairy and beef cattle. Vet Clin N Am Food Animal Pract 1997; 13: 87–106. 10.1016/s0749-0720(15)30366-2 9071748

[bib27] Dusty WN, Jeff WT, Steven BK. Timing of seroconversion and acquisition of positive polymerase chain reaction assay results in calves experimentally infected with bovine leukemia virus. Am J Vet Res 2007; 68: 72–75. 17199421 10.2460/ajvr.68.1.72

[bib28] Nagy DW, Tyler JW, Kleiboeker SB et al. Use of a polymerase chain reaction assay to detect bovine leukosis virus in dairy cattle. J Am Vet Med Assoc 2003; 222: 983–985. 12685791 10.2460/javma.2003.222.983

[bib29] Burgu I, Alkan F, Karaoglu T et al. Control and eradication program of enzootic bovine leucosis (EBL) from selected dairy herds in Turkey. Dtsch Tierarztl Wochenschr 2005; 112: 271–274. 16124702

[bib30] Bartlett PC, Sordillo LM, Byrem TM et al. Options for the control of bovine leukemia virus in dairy cattle. J Am Vet Med Assoc 2014; 244: 914–922. 24697767 10.2460/javma.244.8.914

[bib31] Jacobs RM, Pollar FL, McNab WB et al. Serological survey of bovine syncytial virus in Ontario: associations with bovine leukemia and immunodeficiency-like viruses, production records, and management practices. Can J Vet Res 1995; 59: 271–278. 8548688 PMC1263781

[bib32] Elaine A, Sirois M. Hematology and Heostasis. In: Hendrix M, Sirois HM, editors. Laboratory Procedures for Veterinary Technicians, 5th edn. Elsevier Mosby: Urbana, IL, USA. 2007, pp 33–76.

[bib33] Mirsky ML, Olmstead CA, Da Y et al. The prevalence of proviral bovine leukemia virus in peripheral blood mononuclear cells at two subclinical stages of infection. J Virol 1996; 70: 2178–2183. 8642640 10.1128/jvi.70.4.2178-2183.1996 PMC190056

[bib34] Reichert M, Stec J. Simultaneous use of two primer pairs increases the efficiency of polymerase chain reaction assay in the diagnosis of bovine leukemia virus infection. J Vet Diagn Invest 1999; 11: 543–547. 12968741 10.1177/104063879901100613

[bib35] Mousavi S, Haghparast A, Mohammadi G et al. Prevalence of bovine leukemia virus (BLV) infection in the northeast of Iran. Vet Res Forum 2014; 5: 135–139. 25568707 PMC4279628

[bib36] Uysal A, Yilmaz H, Bilal T et al. Seroprevalence of enzootic bovine leukosis in Trakya district (Marmara region) in Turkey. Prev Vet Med 1998; 37: 121–128. 9879586 10.1016/s0167-5877(98)00108-1

[bib37] Ababneh MM, Al-Rukibat RK, Hananeh WM et al. Detection and molecular characterization of bovine leukemia viruses from Jordan. Arch Virol 2012; 157: 2343–2348. 22914962 10.1007/s00705-012-1447-z

[bib38] Suh GH, Lee JC, Lee CY. Establishment of a bovine leukemia virus-free dairy herd in Korea. J Vet Sci 2005; 6: 227–230. 16131826

[bib39] Schoepf KC, Kapaga AM, Msami HM et al. Serological evidence of the occurrence of enzootic bovine leukosis (EBL) virus infection in cattle in Tanzania. Trop Anim Health Prod 1997; 29: 15–19. 9090010 10.1007/BF02632338

[bib40] Sun WW, Wen-FL, Cong W. *Mycobacterium avium* subspecies paratuberculosis and bovine leukemia virus seroprevalence and associated risk factors in commercial dairy and beef cattle in northern and northeastern China. Biomed Res Int 2015; 2015: 315173. 26504798 10.1155/2015/315173 PMC4609356

[bib41] Rukkwamsuk T, Rungruang S. Seroprevalence of bovine leukemia virus (BLV) infection in pregnant replacement dairy heifers in Saraburi province. Thailand Kasetsart J 2008; 42: 278–281.

[bib42] Morris SD, Myburgh JG, van Vuuren M et al. Serological survey to determine the prevalence of bovine leukaemia virus antibodies in dairy cattle on selected farms in the Gauteng and Mpumalanga governorates. J S Afr Vet Assoc 1996; 67: 146–147. 9120859

[bib43] Yousif AA. Serological study of bovine leukosis in Iraq local and imported cow. Iraqi J Vet Med 1997; 21: 137–142.

[bib44] Dimmock CK, Chung YS, MacKenzie AR. Factors affecting the natural transmission of bovine leukaemia virus infection in Queensland dairy herds. Aust Vet J 1991; 68: 230–233. 1656925 10.1111/j.1751-0813.1991.tb03213.x

[bib45] Moratorio G, Obal G, Dubra A. Phylogenetic analysis of bovine leukemia viruses isolated in South America reveals diversification in seven distinct genotypes. Arch Virol 2010; 155: 481–489. 20169372 10.1007/s00705-010-0606-3

[bib46] Leokadia B, Jacek K, Marzena R et al. Detection genetic diversity among bovine leukemia virus population by single-strand conformational polymorphism analysis. Bull Vet Inst Pulawy 2002; 46: 205–212.

[bib47] Ban J, Gieciova E, Orlik O et al. Use of monoclonal antibodies in an ELISA for the diagnosis of bovine leukaemia virus infection. J Virol Methods 1990; 30: 79–87. 1707889 10.1016/0166-0934(90)90045-h

[bib48] Naif HM, Daniel R, Cougle WG et al. Early detection of bovine leukemia virus by using an enzyme-linked assay for polymerase chain reaction-amplified proviral DNA in experimentally infected cattle. J Clin Microbiol 1992; 30: 675–679. 1313047 10.1128/jcm.30.3.675-679.1992 PMC265131

[bib49] Beirer D, Blankenstein P, Marquardt O et al. [Detection and Identification of different BLV proviruses isolates by PCR, RFLP and DNA sequensis.] Berliner und munchener Tierarztliche Wochenschrift 2000; 114: 252–256.German. 11505797

[bib50] Jacobs RM, Pollari FL, McNab WB et al. Serological survey of bovine syncytialvirus in Ontario: associations with bovine leukemia and immunodeficiency-like viruses, production records, and management practices. Can J Vet Res 1995; 59: 271–278. 8548688 PMC1263781

[bib51] Brandon RB, Naif H, Daniel RCW et al. Early detection of bovine leukosis virus DNA in infected sheep using the polymerase chain reaction. Res Vet Sci 1991; 50: 89–94. 1646474 10.1016/0034-5288(91)90059-w

[bib52] Zaghawa A, Beier D, Abd EL-Rahim IHA. An outbreak of enzootic bovine leukosis in upper egypt: clinical, laboratory and molecular–epidemiological studies. J Vet Med B Infect Dis Vet Public Health 2002; 49: 123–129. 12019942 10.1046/j.1439-0450.2002.00517.x

[bib53] Gutierrez SE, Dolcini GL, Arroyo GH et al. evelopment and evaluation of a highly sensitive and specific blocking enzyme-linked immunosorbent assay and polymerase chain reaction assay for diagnosis of bovine leukemia virus infection in cattle. Am J Vet Res 2001; 10: 1571–1577. 10.2460/ajvr.2001.62.1571 11592321

[bib54] Ooshiro M, Konnai S, Katagiri Y et al. Horizontal transmission of bovine leukemia virus from lymphocytoyic cattle and benegicial effects of insect vector control. Vet Rec 2013; 173: 527. 10.1136/vr.101833 24158325

[bib55] Yamada T, Shigemura H, Ishiguro N et al. Cell infectivity in relation to bovine leukemia virus gp51 and p24 in bovine milk exosomes. PLoS ONE 2013; 8: 77359. 10.1371/journal.pone.0077359 PMC3798320 24146982

